# New epiretinal implant with integrated sensor chips for optical capturing shows a good biocompatibility profile in vitro and in vivo

**DOI:** 10.1186/s12938-021-00938-9

**Published:** 2021-10-12

**Authors:** Kim Schaffrath, Tibor Lohmann, Jan Seifert, Claudia Ingensiep, Pascal Raffelberg, Florian Waschkowski, Reinhard Viga, Rainer Kokozinski, Wilfried Mokwa, Sandra Johnen, Peter Walter

**Affiliations:** 1 Department of Ophthalmology, University Hospital RWTH Aachen, Aachen, Germany; 2 Department of Electronic Components and Circuits, University Duisburg-Essen, Duisburg, Germany; 3 Institute of Materials in Electrical Engineering 1, RWTH Aachen University, Aachen, Germany; 4 Fraunhofer Institute of Microelectronic Circuits and Systems, Duisburg, Germany

**Keywords:** OPTO-EPIRET, Retinal prosthesis, Retinal stimulation, Epiretinal implant, Biocompatibility, Vitreoretinal surgery

## Abstract

**Background:**

Retinal degenerative diseases, e.g., retinitis pigmentosa, cause a severe decline of the visual function up to blindness. Treatment still remains difficult; however, implantation of retinal prostheses can help restoring vision. In this study, the biocompatibility and surgical feasibility of a newly developed epiretinal stimulator (OPTO-EPIRET) was investigated. The previously developed implant was extended by an integrated circuit-based optical capturing, which will enable the immediate conversion of the visual field into stimulation patterns to stimulate retinal ganglion cells.

**Results:**

The biocompatibility of the OPTO-EPIRET was investigated in vitro using the two different cell lines L-929 and R28. Direct and indirect contact were analyzed in terms of cell proliferation, cell viability, and gene expression. The surgical feasibility was initially tested by implanting the OPTO-EPIRET in cadaveric rabbit eyes. Afterwards, inactive devices were implanted in six rabbits for feasibility and biocompatibility testings in vivo. In follow-up controls (1–12 weeks post-surgery), the eyes were examined using fundoscopy and optical coherence tomography. After finalization, histological examination was performed to analyze the retinal structure. Regarding the in vitro biocompatibility, no significant influence on cell viability was detected (L929: < 1.3% dead cells; R-28: < 0.8% dead cells). The surgery, which comprised phacoemulsification, vitrectomy, and implantation of the OPTO-EPIRET through a 9–10 mm corneal incision, was successfully established. The implant was fixated with a retinal tack. Vitreal hemorrhage or retinal tearing occurred as main adverse effects. Transitional corneal edema caused difficulties in post-surgical imaging.

**Conclusions:**

The OPTO-EPIRET stimulator showed a good biocompatibility profile in vitro. Furthermore, the implantation surgery was shown to be feasible. However, further design optimization steps are necessary to avoid intra- and postoperative complications. Overall, the OPTO-EPIRET will allow for a wide visual field and good visual acuity due to a high density of electrodes in the central retina.

## Background

Retinitis pigmentosa (RP) is an inherited neurodegenerative disease of the retina and leads to night blindness in early stage, followed by restriction of the visual field, reduction in contrast and color vision, reduced visual acuity and in end stage to blindness because of progressive photoreceptor degeneration [1–3]. Photoreceptors can be divided into rods and cones. Cones are located mainly in the macula, are sensitive to color, and important for precise acuity in daylight. Rods are located throughout the retina and are sensitive to light mediating achromatic vision in starlight or moonlight. In RP, rods degenerate first, causing the restrictive visual field; only in late stages, cones are also affected [3]. Mutations in more than 70 genes encoding for basic visual processes can cause a progressive degeneration of photoreceptors and about 1 out of 4000 of the population is affected by RP [3]. Treatment is complex and difficult and still not solved. It comprises symptomatic therapy, visual prostheses to replace the photoreceptors’ function, and one available gene therapy approach, which addresses biallelic *RPE65* gene mutations in RP patients (Luxturna®, voretigene neparvovec-rzyl) [1]. Prostheses can be implanted at different places. One approach is epiretinal, which is represented by the former commercially available Argus II® Retinal Prosthesis System (Second Sight Medical Products, USA) or IRIS II (Intelligent Retinal Implant System, Pixium Vision, France) [4, 5]. The Alpha AMS implant (Retina Implant AG, Germany) or the PRIMA implant (Pixium Vision, France) are representatives for the subretinal approach [6, 7]. An Australian and a Japanese group work with suprachoroidal implants [8, 9]. Another possibility to stimulate the visual pathway is a cortical implant like the Orion (Second Sight) [10]. The results of approved existing implants, which were used in clinical settings, e.g., Argus II®, IRIS II or Alpha AMS, are limited and all of them are no longer commercially available. The prostheses could elicit phosphenes and improve performance in some visual tasks, but did not restore normal vision [10]. Therefore, the question of improving retinal prostheses is still not solved and needs to be addressed.

According to epiretinal prostheses, a longstanding experience is described in literature (e.g., EPIRET3 [11], VLARS [12], POLYRETINA [13]). Different numbers of electrodes and varying sizes of the implant were investigated. Recently published, a very large electrode array for retinal stimulation (VLARS) was explored in vivo to obtain a large visual angle with a high number of electrodes (250 electrodes) on top of the flexible foil [12, 14]. We showed that the implantation surgery was feasible, but difficult due to the size of the device. Therefore, we worked on the development of a new epiretinal prosthesis with a slightly reduced diameter and with Application Specific Integrated Circuits (ASICs) with integrated photosensors [15]. These newly integrated sensorchips will allow for image capturing and immediate conversion of the visual field into stimulation patterns within the eye. Therefore, the images that normally fall directly onto the retina, are recorded by the photosensors at the backside of the Integrated Circuit (IC). The optical information is then converted by the IC into appropriate stimulation pulses that are forwarded through a flexible foil to the microelectrodes on the backside of the foil to stimulate the remaining retinal ganglion cells (RGCs).

Furthermore, this approach holds promise in preventing local adaptation processes in the retina by using the still intact microsaccades. To restore a good visual acuity, a high electrode density in the central OPTO-EPIRET array is important with a connection of one photodiode to one stimulation electrode for the cone-driven channel. For the peripheral retina, several parallel-connected photodiodes should be connected to one stimulation electrode to simulate the convergent rod channel. However, as the primary focus was on the testing of the biocompatibility and feasibility, only a representative small number of electrodes were mounted on the implanted OPTO-EPIRET device.

We focused on in vitro and in vivo biocompatibility as well as on the surgical feasibility of the newly developed device. The in vitro biocompatibility profile comprising direct and indirect contact corresponding to the standard ISO 10993 “biological evaluation of medical devices,” parts 5 and 12 was firstly investigated [16]. The experiments were performed with the sensitive cell line L-929 [17] and with R28 cells, which result from the immortalization of postnatal day 6 rat retina and represent a retinal progenitor cell line with both neuronal and retinal characteristics [16, 18–20]. Secondly, we performed and optimized the surgical implantation procedure in cadaveric rabbit eyes. Thirdly, implantation of the epiretinal device was carried out in six rabbits, which were monitored and examined over a 12-week follow-up. After finalization, the analysis of the in vivo biocompatibility profile as well as histological investigations were conducted.

## Results

### In vitro biocompatibility

#### Effects of extractive media on cell survival

**Fig. 1:**
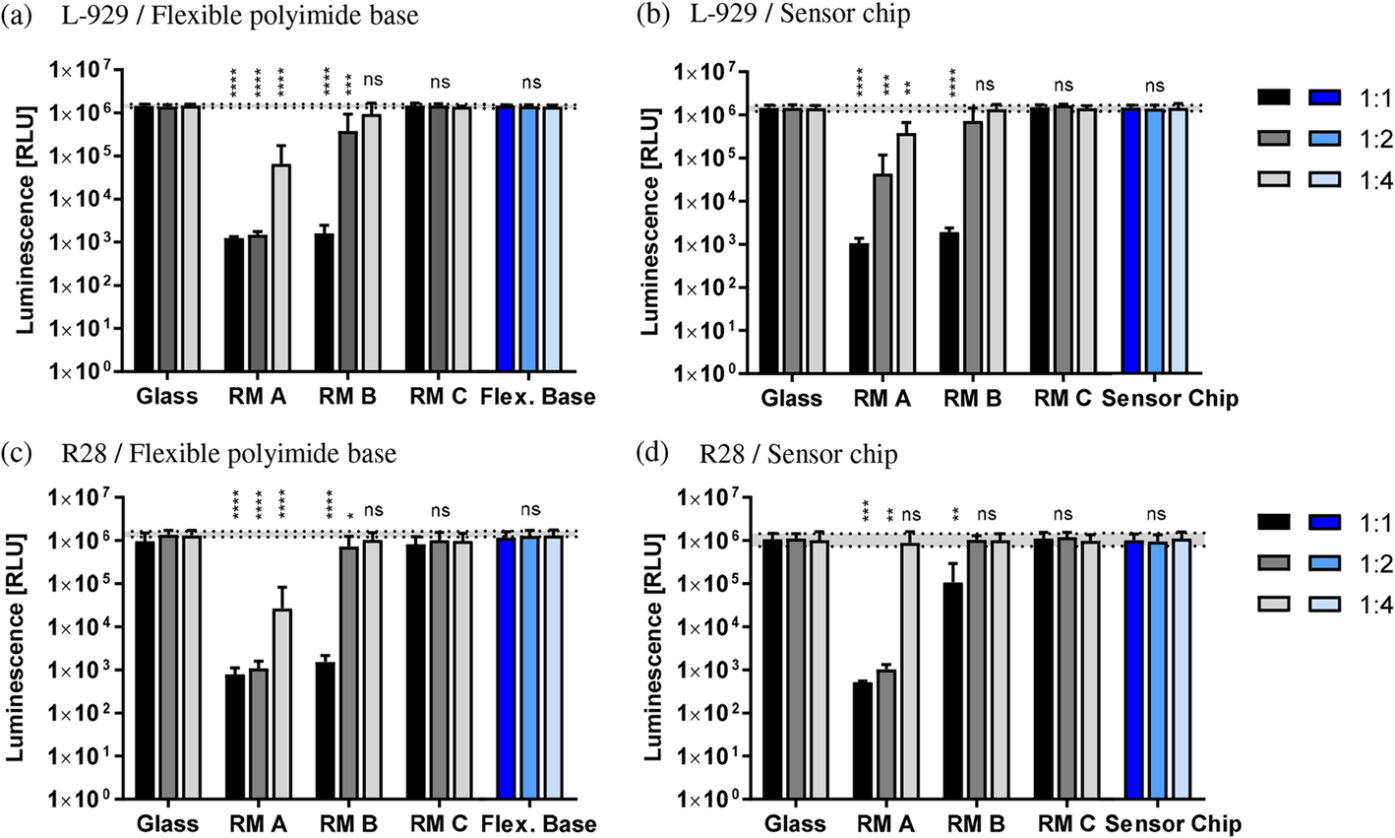
Effects of extractive media on cell survival. Survival rates of L-929 (**a**, **b**) and R28 (**c**, **d**) cells were analyzed in cultures incubated with increasing dilutions (1:1, 1:2, 1:4) of extractive media obtained from certified positive (RM A, RM B) and negative (RM C) reference materials as well as from negative (glass) controls and from the test structures; **a**, **c** flexible polyimide base; **b**, **d** sensor chip. The dotted lines designate mean values of the glass approaches (negative control). The results were compared to glass. Bars were calculated as mean ± SD. One-way ANOVA with Dunnett’s post hoc test was performed; **p < 0.01, ***p < 0.001, ****p < 0.0001. Flexible polyimide base: for L-929 cells: *n* = 4 individual experiments, for R28 cells: *n* = 6. Sensor chips: for L-929 and R28 cells: *n* = 8

For both cell lines, L-929 and R28, incubation with the extractive media of either the negative control reference material (RM) C or glass showed a constant luminescence at each applied dilution and no cytotoxic effect (see Fig. 1). For both L-929 and R28 cells nondiluted extractive media of positive control RM A and RM B reduced the luminescence for more than 99.9%, except for RM B and R28 cells with nondiluted extractive media of the sensor chip (> 90%). The decreasing reduction of luminescence is correlated to the higher dilution steps and the subsequent lower toxicity. The differences between the reduced luminescence of RM A and RM B extractive media confirmed the higher level of cytotoxicity of RM A. However, the tested flexible polyimide bases and the sensor chips showed no reduction of the luminescence for all dilution steps, indicating that there was no cytotoxicity.

#### Effects of direct contact on cell viability

**Fig. 2:**
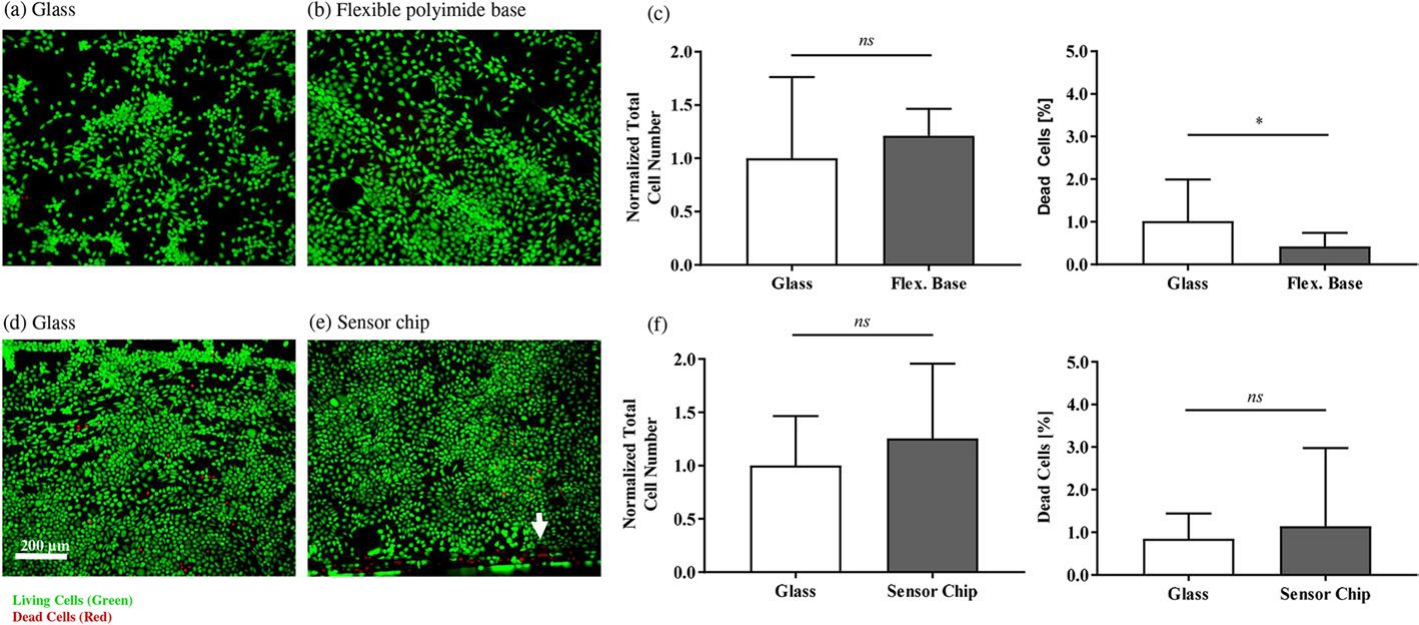
Viability of L-929 directly cultivated on glass and on flexible polyimide bases. **a**, **b**, **d**, **e** Fluorescence microscopy allowed for differentiation between vital (green) and dead (red) L-929 cells 72 h after seeding. **a**–**c** Flexible polyimide base; **d**–**f** sensor chip. Note the white arrow in e showing dead cells on the edge of the sensor chip. **c**, **f** The left graphs show the normalized total cell number. The right graphs present the quantity of dead cells as percentage of the total cell number. For each substrate, 3 to 6 randomly selected image sections were analyzed. The total cell amount was normalized to the total cell amount on glass. The results were compared to glass. Bars were calculated as mean ± SD (unpaired *t*-test; ns: not significant, *p < 0.05; flexible polyimide bases: *n* = 4 individual experiments; sensor chips: *n* = 8)

**Fig. 3:**
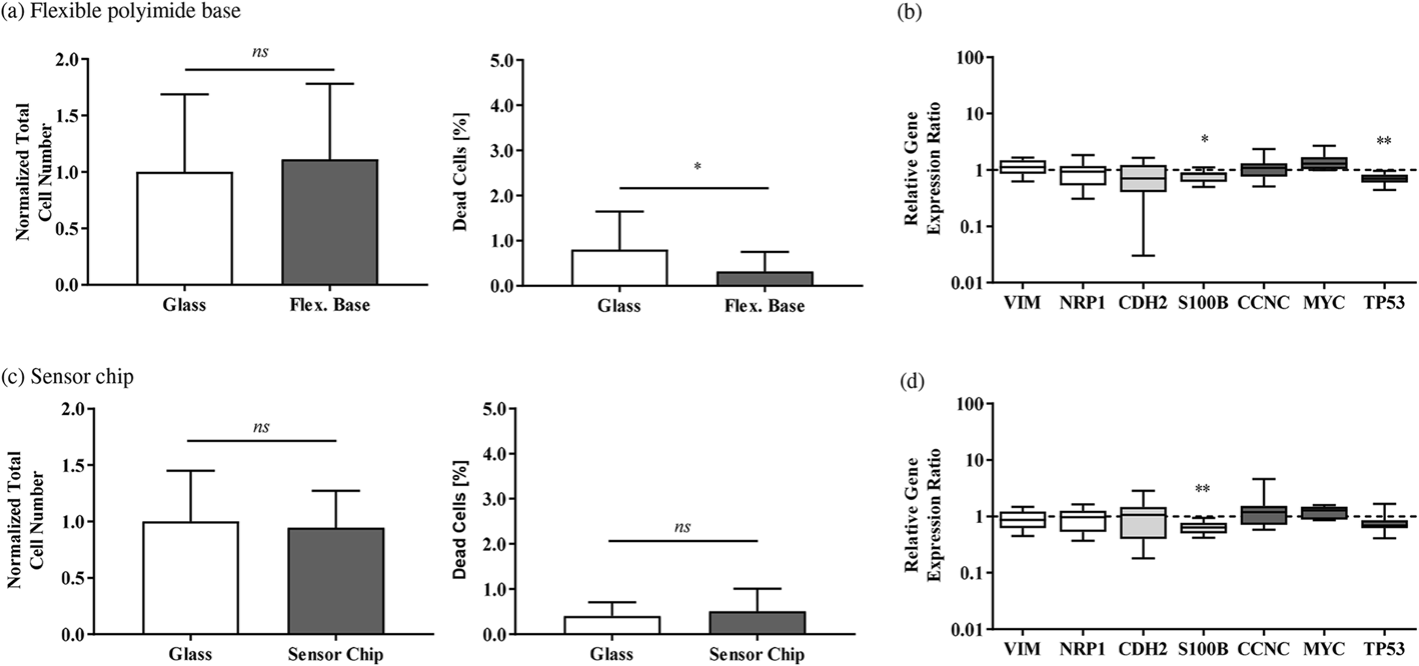
Direct contact analysis and gene expression in R28 cells. **a**, **b** Results of flexible polyimide bases, **c** and **d** results of sensor chips. **a**, **c** The left graph shows the normalized total R28 cell number for flexible polyimide base and sensor chip, respectively. The right graph presents the quantity of dead cells of the total cell number for flexible polyimide base and sensor chip, respectively. For each substrate, 3 to 6 randomly selected image sections (original magnification, ×100) were analyzed. The total cell amount was normalized to the total cell amount on glass. The results were compared to glass. Bars were calculated as mean ± SD (unpaired *t*-test; *p < 0.05, *ns* no significance; flexible polyimide bases: *n* = 5; sensor chips: *n* = 8). **b**, **d** Real-time PCR was performed with cDNA templates of R28 cells to quantify the expression of different genes involved in the cell cycle and representing neuronal/glial and retinal markers. Using the comparative CT (2^−ΔΔCT^) method, the relative gene expression ratio of cells cultivated on glass was set to 1. Regarding cultivation on the different test structures, values > 1 denote upregulation and values < 1 denote downregulation of gene expression. Each column represents the median, maximum, minimum, and the 50th percentile of the data for 4 distinct LightCycler runs (one sample two-tailed *t*-test; *p < 0.05, **p < 0.01; white bars: retinal marker; light grey bars: neuronal marker; dark grey bars: cell cycle/oncogenes; *n* = 4 individual experiments)

Both cell lines, L-929 and R28, grew on glass and on the test structures (see Figs. 2 and 3). There was no significant difference between the total normalized cell number on glass and the test structures (see Figs. 2c, f and 3a, c, left graph). For all structures, less than 3% dead cells were observed. For the flexible polyimide bases, a significantly smaller amount of dead cells was measured on the test structures than on glass (L-929 cells on test structure: 0.4 ± 0.3% vs. on glass 1.2 ± 1.2%; R28 cells on test structure: 0.2 ± 0.1% vs. on glass 0.7 ± 0.9%). Regarding the sensor chips, there was no significant difference between the percentage of dead cells on glass and on the test structures (L-929 cells on test structure: 1.1 ± 1.8% vs. on glass 0.9 ± 0.6%; R28 cells on test structure: 0.5 ± 0.5% vs. on glass 0.4 ± 0.3%). We observed, that most of the dead cells appeared close to the sharp edges of the sensor chips (see Fig. 2e). Overall, negligible cytotoxicity and reduction in cell viability was seen. Regarding the growth properties of the R28 cells, a formation in clusters was noticed (data not shown), whereas L-929 cells grew without any cluster formation (see Fig. 2a, b, d, e).

#### Gene expression profile of R28 cells after cultivation on the test structures

Regarding the gene expression profile, most of the analyzed genes did not show any significant changes compared to gene expression after cultivation on glass (see Fig. 3b, d, relative gene expression ratio of cells cultivated on glass was set to 1). The genes encoding for the retinal markers VIM (vimentin) and NRP1 (neuropilin-1) as well as the neuronal marker CDH2 (cadherin-2) and the cell cycle markers/oncogenes CCNC (cyelin-C) and MYC (myc proto-oncogene protein) showed an unaltered gene expression profile. Only a slight decrease in the gene expression of S100B (protein S100-A10) (0.80 ± 0.20) and TP53 (cellular tumor antigen p53) (0.71 ± 0.16) was observed after cultivation on the flexible polyimide bases, and of S100B (0.65 ± 0.17) for the sensor chips.

### In vivo biocompatibility

#### Performing surgery

**Fig. 4:**
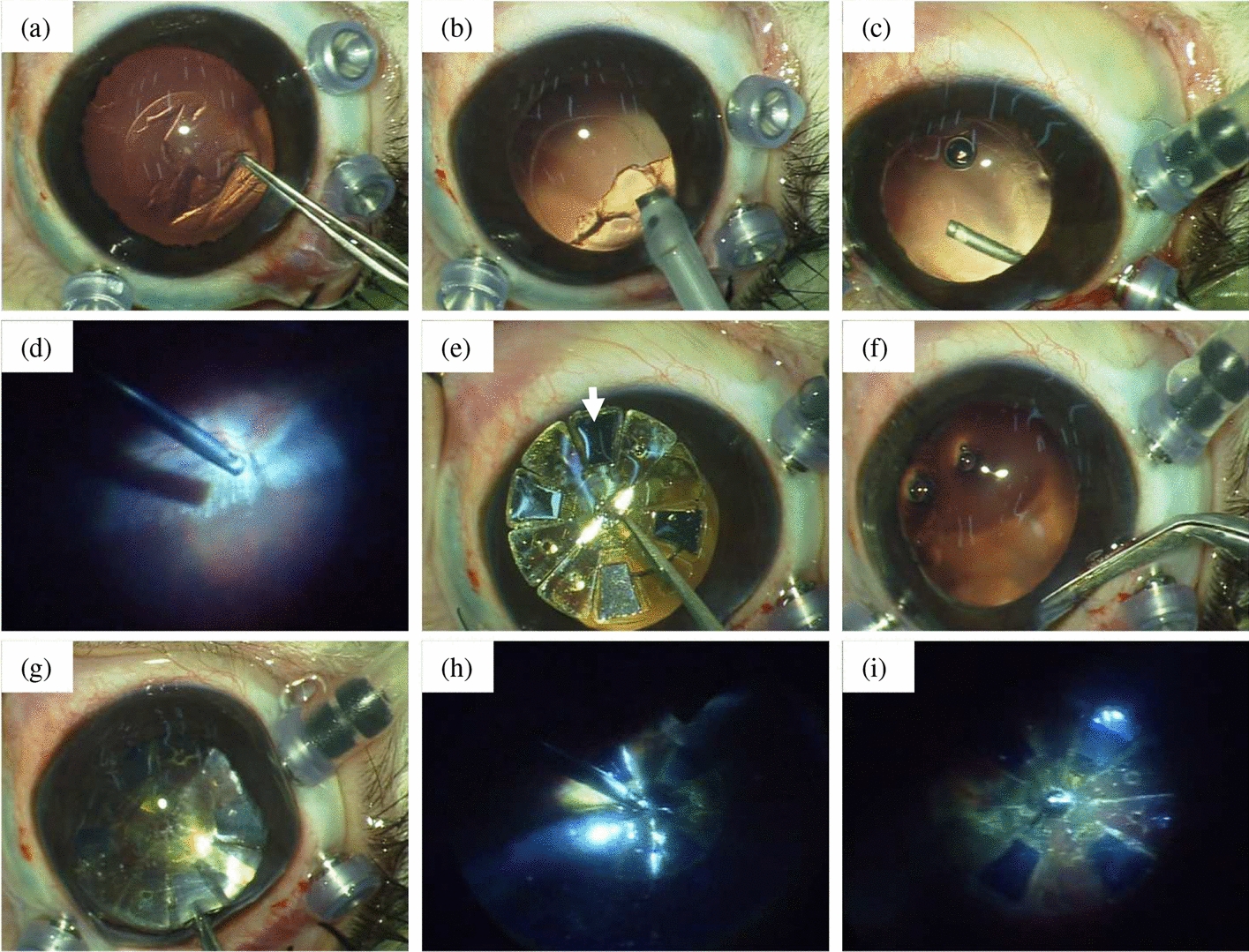
Implantation surgery of the OPTO-EPIRET array in a rabbit eye. **a** Anterior capsulorhexis; **b** phacoemulsification; **c** posterior capsulorhexis; **d** pars-plana vitrectomy; **e** dummy OPTO-EPIRET array; **f** corneal incision; **g** OPTO-EPIRET in the anterior chamber, manipulated with surgical forceps; **h** fixation of the OPTO-EPIRET on the retinal pole using a titanium retinal tack; **i** final position of the array at the end of the implantation surgery

The implantation surgery of the dummy OPTO-EPIRET array was tested at first in cadaveric rabbit eyes to improve the surgical steps and the device handling. The best way to insert the array into the anterior chamber was established. This resulted in creating a big corneal incision parallel to the limbus to get access to the anterior chamber (see Fig. 4f). Folding the array, as it was performed with the VLARS array [12], was difficult due to the sensor chips’ stiffness and, hence, not useful. After establishing the optimal surgical procedure steps, the non-folded dummy OPTO-EPIRET array was inserted through a 9–10 mm corneal incision into the anterior chamber (see Fig. 4g) und then forwarded to the vitreous cavity in vivo. The next step to move the array to the right position without touching the retina was crucial as well. Therefore, the perfluocarbon liquid (PFCL) bubble was used as a cushion for the array. By slowly removing the PFCL bubble, the implant was lowered towards the retina. When the array was placed at the right position on the central retina besides the optic disc, it was fixated with a retinal tack (see Fig. 4h). Finally, suturing all cuts precisely was another critical step.

In all of the six implantations, lensectomy and vitrectomy were performed without any complications. The surgery procedure took 1 h and 40 min in mean, time was decreasing over the course of the study. In two cases, a sensor chip detached. In one case, a vitreal hemorrhage occurred, but this was staunched during surgery. In three cases, retinal tearing was described. After each implantation, a mild corneal edema at the side of the corneal incision arose. At the end of the surgery, the tackling of the dummy OPTO-EPIRET array showed up difficult, but was successfully performed in all implantations. All adverse events are summarized in Table 1.

**Table 1:** Summary of the chronic implantations of the OPTO-EPIRET device in rabbits

Animal no.	Duration of surgery (h:min)	Adverse events during surgery	Follow-up adverse events	OCT-imaging	Open-sky imaging
1	2:20	Dislocation of sensor chip, mild vitreal hemorrhage, difficult fixation using retinal tack, corneal edema	Corneal edema with neovascularization (see Fig. 6)	Retina: good quality, parts of implant with distance to retina; cornea: good quality, corneal edema (see Fig. 6)	Retinal detachment (superior hemisphere), mild gliosis on array
2	1:45	Retinal tear (inferior hemisphere), difficult fixation using retinal tack, corneal edema	Slight corneal edema	Retina: acceptable quality, gliosis, pricking with one wing into retina; cornea: good quality, corneal scar	Slight retinal detachment
3	1:20	Retinal tear (temporal hemisphere), difficult fixation using retinal tack, corneal edema	Fibrinous reaction (control at week 5 no fibrin), subluxation of the retinal tack, slight corneal edema	Retina: good quality, gliosis, central good contact (see Fig. 7), at least one wing deviated; cornea: good quality, corneal edema	Insufficient fixation of retinal tack, two wings deviated, retinal detachment
4	1:35	Retinal tear (central retina), difficult fixation using retinal tack, corneal edema	Ectropion, vitreal hemorrhage, low IOP, severe vascularization of corneal edema, suspected dislocation of the array, premature finalization after 3 weeks (d23)	Retina: not good quality; cornea: not performed	Vitreal hemorrhage, gliosis
5	1:10	Corneal edema	Very slight corneal edema	Retina: good quality, partly no good contact between array and retina, gliosis, pricking with one wing into retina (see Fig. 7); cornea: good quality, central normal corneal thickness, just slight edema peripheral (see Fig. 6)	Gliosis on array
6	1:45	Dislocation of one sensor chip, corneal edema	Weight loss, slight corneal edema, week 2 breathing arrest under anesthesia (suspicion of heart problems)	Retina: good quality, good contact (see Fig. 7), not good contact of one wing, gliosis; cornea: good quality, no corneal edema	Gliosis on array, dislocation of one sensor chip, good contact, retinal detachment

*d* day, *IOP* intraocular pressure

In summary, the surgery, which comprises phacoemulsification, vitrectomy and implantation of the OPTO-EPIRET stimulator through a 9–10 mm corneal incision, was successfully established. The array was fixated on the posterior pole with a retinal tack. Vitreal hemorrhage, retinal tearing and corneal edema occurred as main adverse effects.

#### Post-surgery follow-up

Five of six rabbits fulfilled the clinical follow-up examinations over the period of 12 weeks. However, one animal (no. 4) had to be premature finalized after 3 weeks due to vitreal hemorrhage, low intraocular pressure (IOP), ectropion and a suspected dislocation of the array. Another animal (no. 6) showed a breathing arrest under anesthesia and a loss of weight after anesthesia. As a consequence, follow-up at weeks 4 and 8 involved only clinical examination without further anesthesia. For a clear overview, all adverse events are summarized in Table 1.

**Fig. 5:**
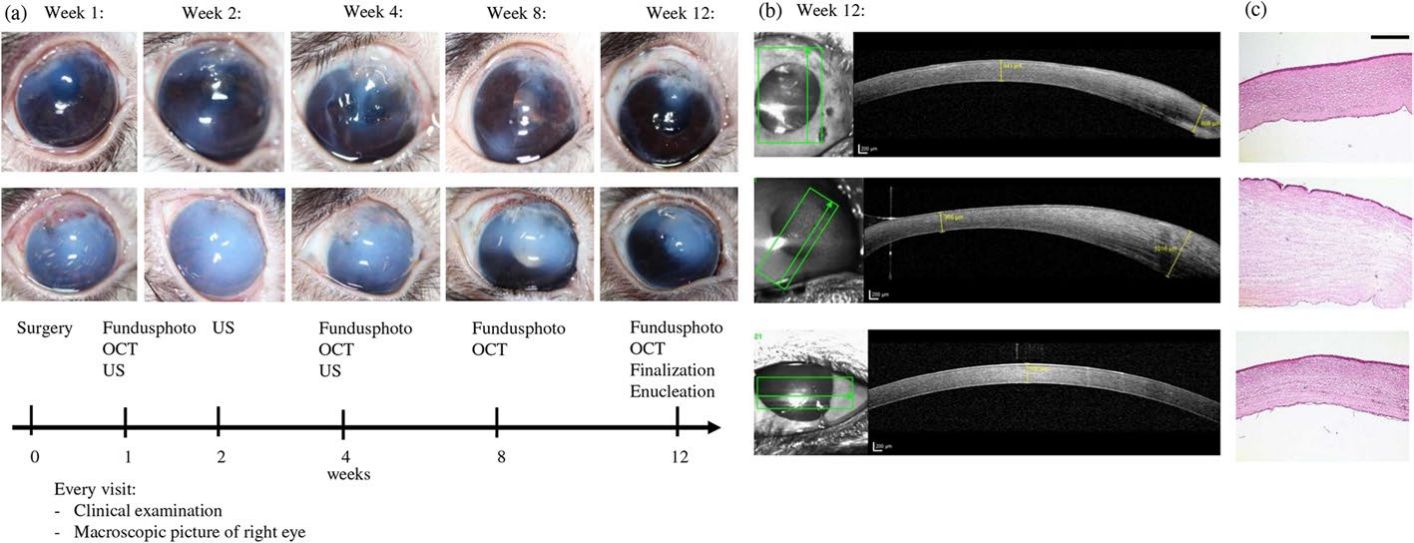
**a** Clinical follow-up examination and photography of the implanted right eye after the implantation surgery; upper row: animal no. 5. Week 1 and 2: corneal edema, corneal incision sufficiently sutured; weeks 4 to 12: regression of the corneal edema under topical treatment with antibiotics and corticosteroids. Development of corneal scarring, fibrosis. Central opacity given at all times. Lower row: animal no. 1, week 1 to 4: corneal edema and vascularization; weeks 9 to 12: regression of corneal edema under topical treatment with antibiotics and corticosteroids. Central opacity not given at all times. *US* ultrasound, *OCT* optical coherence tomography. **b** OCT imaging of the cornea after implanting an inactive OPTO-EPIRET array into a rabbit eye. Note the corneal thickness due to edema (yellow caliper; first OCT: animal no. 5, week 12; second OCT: animal no. 1, week 12; last OCT: control of left non-implanted eye, animal no. 5; **c** hematoxylin and eosin (H&E) staining of the cornea, top: cornea of implanted eye, animal no. 5, middle: cornea of implanted eye, animal no. 1, bottom: control left eye (animal no. 5); scale bar represents 500 µm

**Fig. 6:**
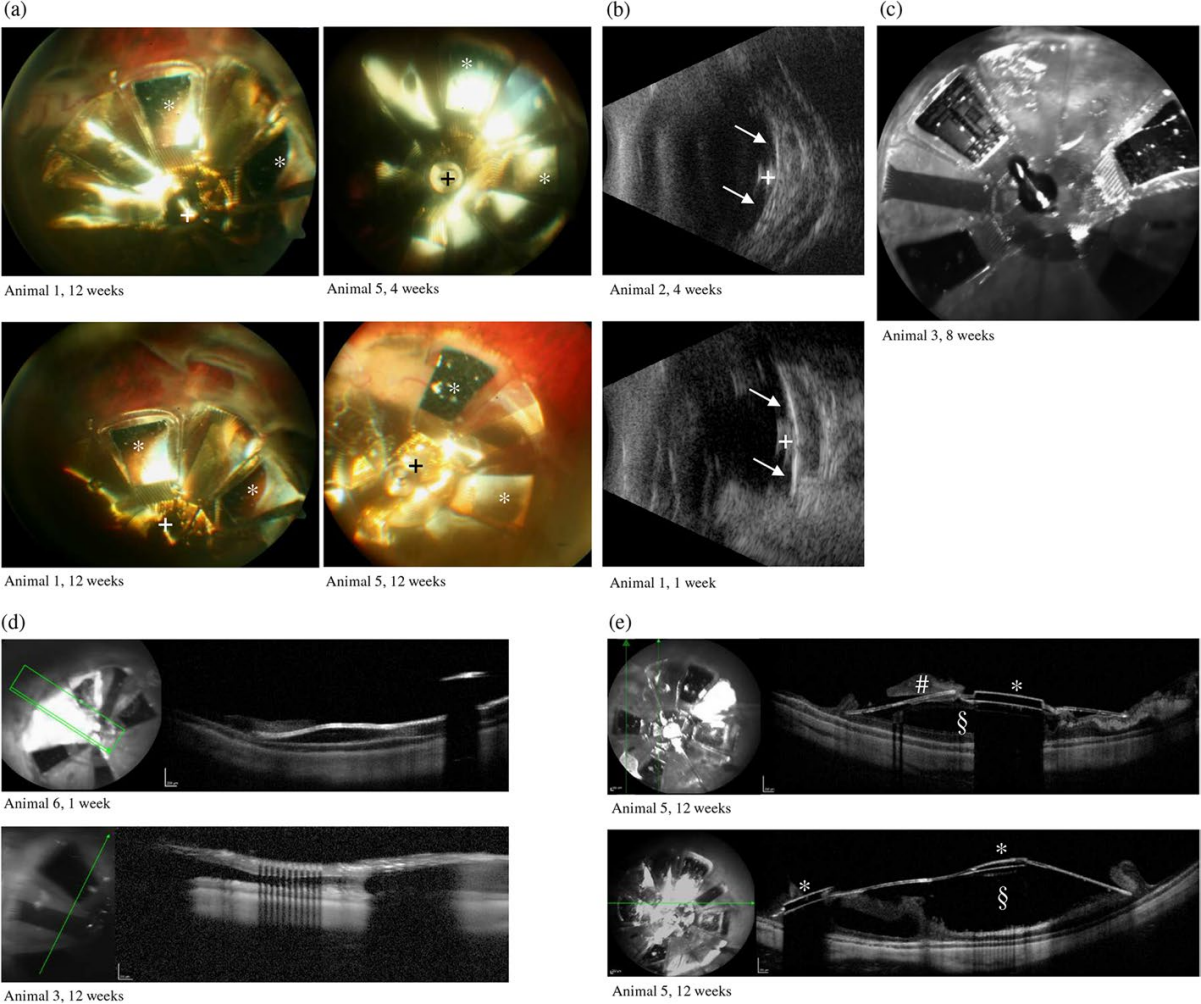
Images of the implanted right eye of different animals at different points of time. **a** Fundus photographies; **b** ultrasound images; **c**–**e** OCT images; **d** close approximation between the array and retina; **e** considerable gap between the array and retina; arrow: ultrasound reflex of wings; plus sign: retinal tack, asterisk: sensor chip on array; section sign: gap between array and retina; number sign: gliotic tissue

None of the animals showed a sign of severe intraocular inflammation or endophthalmitis over the period of 12 weeks follow-up. In one case, a fibrinous reaction in the anterior chamber appeared and was treated with topical corticosteroids (animal no. 3). Transitional corneal edema was observed in all animals with different intensities (see Fig. 5) and showed regression under topical application of corticosteroids over time. The thickness of the swollen cornea could be quantitatively measured by optical coherence tomography (OCT) images (up to 1000 µm instead of the normal thickness of 400 µm) and was still present 12 weeks postoperatively in animals no. 1–3 (see Fig. 5). The corneal edema caused difficulties in post-surgical imaging. If the corneal edema was too pronounced to achieve a high-quality retinal OCT or fundus imaging to evaluate the alignment of the array, ultrasound imaging was performed (see Fig. 6b). In four eyes a good fixation of the array was achieved by the retinal tack. However, in five cases either a deviated wing or a wing pricking into the retina was detected (see Fig. 6e). Gliotic tissue on the array as well as close to the array was detected in four eyes. In one case, a subluxation of the central retinal tack occurred, but the array remained in the correct position (animal no. 3). Animal no. 6 showed a retinal detachment during the last follow-up examination.

#### Open-sky evaluation and histological analysis

After finalization of the rabbits and before the retinae were isolated, embedded, stained, and fixated, the open-sky situation was evaluated. We recognized four cases of retinal detachment (animals no. 1, 2, 3, and 6). The cause and time of retinal detachment detected during dissection could not be determined in every case. Although a retinal tear occurred during the implantation in the animals no. 2, 3, and 4, a retinal detachment did not appear intraoperatively in any animal.

**Fig. 7:**
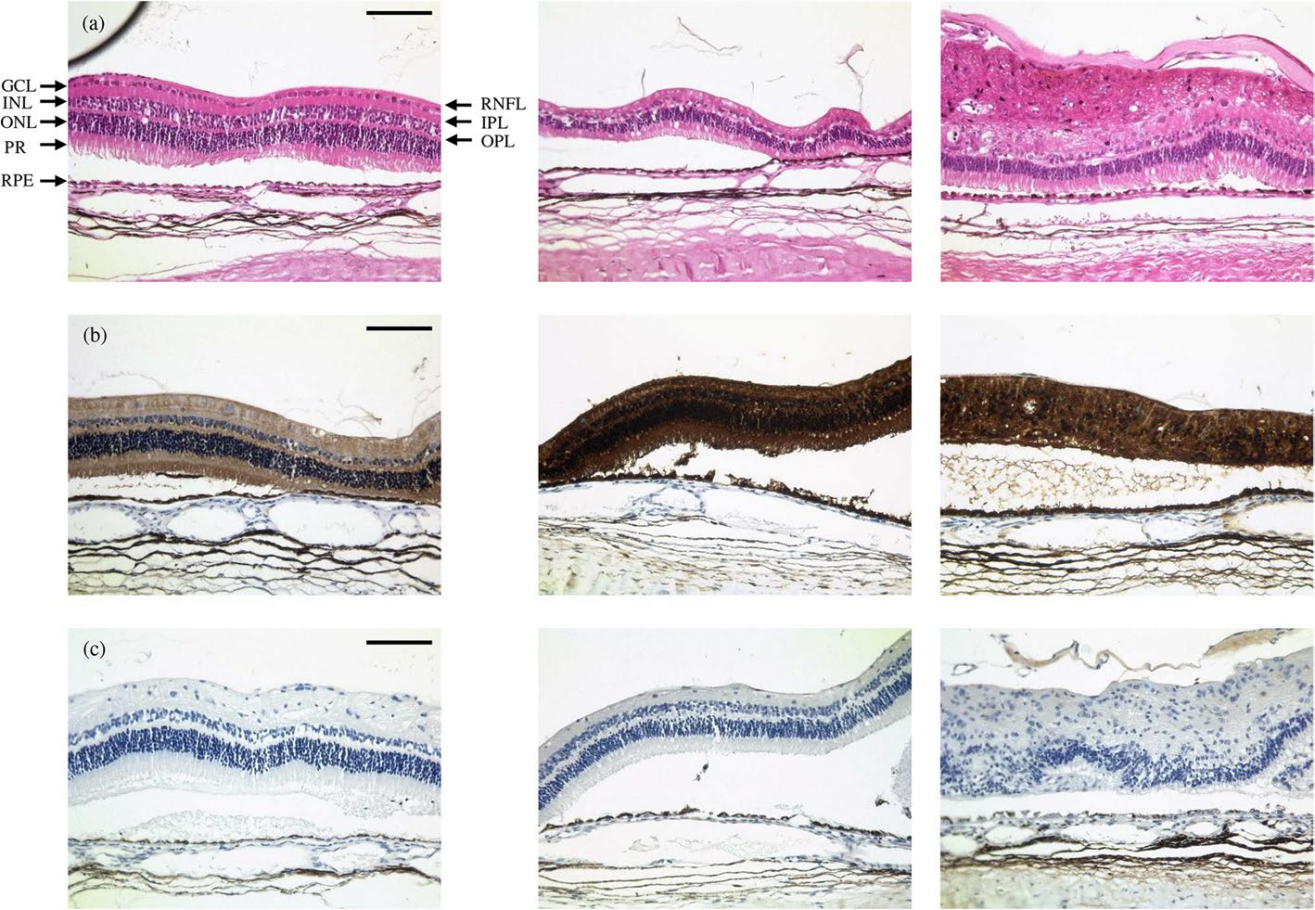
Histological examination of retinal tissue after 12 weeks, animal no. 5. Left: control left eye, middle/right: area under the OPTO-EPIRET; **a** H&E staining, **b** GFAP staining, **c** CD45 staining. Scale bars represent 100 µm. *RNFL* retinal nerve fiber layer, *GCL* ganglion cell layer, *IPL* inner plexiform layer, *INL* inner nuclear layer, *OPL* outer plexiform layer, *ONL* outer nuclear layer, *PR* photoreceptors, *RPE* retinal pigment epithelium

Correlating with the aforementioned retinal damage due to deviated wings and as a consequence of pricking into the retinal tissue, some H&E-stained samples showed damaged retinae (data not shown). However, samples of undamaged retinae showed mostly intact retinal layering, even in the area beneath the implant (see Fig. 7a). In some cases, the retinal layering was disorganized and the retinal thickness was reduced. In those cases, cell count in the outer nuclear layer was often reduced. Additionally, in epiretinal gliosis as well as an increase in eosinophilia was detectable in a few H&E slices (see Fig. 7a). However, signs of significant inflammation were not detected. Immunostaining showed an increased GFAP (glial fibrillary acidic protein)-activity across all implanted eyes (see Fig. 7b), whereas CD45-staining showed no aggregation of leucocytes, i.e., CD45-positive cells (see Fig. 7c).

## Discussion

The basic idea of OPTO-EPIRET is the integration of photodiodes in a retinal implant to avoid the use of any external camera and to implement targeted eye movements. Hence, the targeted image falls onto the image detector and stimulation of the RGCs is topographically accurate at the correct retinal position. Moreover, the use of physiological microsaccades prevents an attenuation of perception.

Overall, our study was pointing at evaluating the in vitro and in vivo biocompatibility of the newly developed OPTO-EPIRET approach as well as the feasibility of the surgical procedure. Neither contact with material extracts nor seeding of test cells onto the structures revealed cell-toxic effects, and also the expression profile of different genes involved in cell cycle and representing retinal or neuronal/glial markers showed any relevant changes. For the in vitro biocompatibility testings two different cell lines were used, since cell lines enable the sustained supply of large quantities of stable cells with consistent cellular properties. The murine fibroblast cell line L-929 is used in many standardized tests to determine the cytotoxicity and biocompatibility of various materials, as stated in the DIN EN ISO guidelines for the biological evaluation of medical devices [21–23]. In addition to this general screening, we considered the actual target site of the implants by using the R28 cell line. The retinal progenitor cell line R28 was established in the late 1990s starting from a 6-day-old immortalized rat retinal culture. R28 cells express the glial cell markers GFAP, S-100, and vimentin and respond to neurotransmitters and light stimulation, suggesting the presence of different retinal neurotransmitter receptors [18, 19, 24, 25].

After cultivation of the cell lines on glass, we determined a higher number of dead cells than on the flexible polyimide bases. This is most likely due to a “poorer/reduced” adhesion of the cells. In normal cell cultures, cells are cultivated on glass or plastic. However, sometimes adhesion of the cells to the glass/plastic surface is not ideal, so that the surfaces are often coated with glycoproteins such as laminin or collagen. However, regarding the biocompatibility testing, an untreated surface should be used as control.

Analysis of the direct cell contact was carried out deliberately without encapsulation of the silicon-based sensor chip, in order to detect potentially cytotoxic effect of the chip itself. The good biocompatibility profile of parylene C which was used for later encapsulation was already proofed [12, 26].

In the open-sky evaluation, some cases of retinal detachment were observed without a clear indication regarding cause and time. Since dissection of the eyes was done immediately after enucleation without previous embedding, the eyes were flaccid so that movement which could lead to retinal detachment was unavoidable.

The immunohistological investigations showed an increased glial reaction, increased eosinophilia, and a higher GFAP activity in the retinal tissue beneath the implanted device. This could be linked to the vitreoretinal surgical procedure of this magnitude [12, 27]. Moreover, a pressure-induced retinal atrophy was observed due to the lack-based fixation process. However, the immunohistological results proved that there was no significant increase in CD45-positive cells, meaning no immigration of inflammatory cells into the retina as immune reaction [28]. Therefore, we can assume that further improvements in the design of such structures are necessary, but that they are fundamentally suitable for stimulating the retina and enable new ways of transmitting signals and energy into a retinal implant.

Regarding the in vivo biocompatibility, we found a considerable number of adverse events and complications related to the relation between the size of the eye and the implant on the one hand and to the sharp-edged device structure on the other hand leading to retinal breaks. The standard implantation procedure itself was straight forward and feasible. Nevertheless, the device was somewhat stiffer than a purely flexible array, thus requiring a larger surgical approach for implantation. Due to the large corneal incision, corneal opacities and edema occurred frequently as a main side effect. Furthermore and due to the stiffness, there was a higher risk of iatrogenic retinal tears which could result in retinal detachment. Another difficulty was the fixation of the array which resulted in insufficient contact between the wings of the device and the retinal surface. Hence, the stimulation efficiency of the RGCs could be reduced. A great challenge yet to conquer is to create structures comprising smoother edges, showing a higher degree of flexibility, as well as sharper fixation tacks to reduce the amount of force necessary for insertion into the retina. To optimize the retina-array contact area, a pre-curved design of the device would support a better alignment, and the future use of bioadhesives or thermosensitives instead of retinal tacks could further reduce the gliotic reaction as well as avoid the traumatic fixation [29–32]. Overall, we assume that further development of the array regarding an improved flexibility and a less traumatic way of fixation combined with an advanced surgerical implantation procedure supported by a shooter or port system could be possible optimization steps. However, the VLARS study already showed that an implantation cone was not beneficial [12].

For biocompatibility testing, an OPTO-EPIRET device with a reduced number of electrodes was used. The electrodes on the central chip were missing and only four ASICs on the wings were assembled, due to the complexity of manufacturing and connecting the form-aligned complementary metal–oxide–semiconductor (CMOS) chips on the array. Following the positive results of the biocompatibility testing, the number of electrodes will be expanded. The missing central chip will be added, 113 electrodes will be placed on each wing and 72 electrodes in the center which will lead to a total electrode number of 1089. Overall, the OPTO-EPIRET restores a theoretical visual angle of approximately 30° in a normal-sized human eye [14, 33], resulting in safely navigating through an observed environment [12, 34]. Nevertheless, the theoretical visual angle of VLARS or POLYRETINA comprises a larger visual angle, 37.6° and 43°, respectively [12, 13, 35].

In 2018, Ferlauto and colleagues presented a similar and promising photovoltaic epiretinal approach called POLYRETINA [13]. This flexible, foldable and self-opening array with an hemispherical shape mounts 2215 photovoltaic stimulating pixels and can be implanted via a 6.5-mm large scleral incision. Just recently, the photovoltaic pixel number was enlarged to 10,498 pixels [35]. POLYRETINA overcomes most of the above-mentioned challenges of OPTO-EPIRET, combines an increased size of the array and a huge number of stimulation pixels to increase both visual acuity and visual field size. But, so far, the auspicious in vitro and ex vivo results still have to be transferred and approved in an in vivo study [13, 35].

Compared to the conventional epiretinal implants that were already used in clinical settings, e.g., ARGUS II®, the new OPTO-EPIRET approach will integrate the image capturing, processing and stimulating within the chip thus rendering the external power supply or glasses unnecessary, whereas the ARGUS II® is dependent on the external parts. In addition, the integrated approach enables targeted eye movements and topographically accurate retinal stimulation.

When comparing the epiretinal with the subretinal photovoltaic approach, e.g., alpha AMS, PRIMA, the epiretinal one shows the advantage of good fixation of wide-field implants, whereas large subretinal approaches can encounter difficulties in the surgical subretinal placement and, hence, representing a higher risk of retinal detachment [8, 10, 35].

All retinal prostheses still do not satisfy the patients’ and researchers’ expectations of restoring normal vision. Therefore, they all have to increase the visual outcome. Furthermore, they also did not consider the degeneration processes within the retina nor the changes in the visual pathway. Nanotechnologies, e.g., nanomaterials for electrodes, organic polymer materials or optimized phased array emitters, can be used to optimize the hardware [36–43]. This will increase the electrode count and lead to a more precise image processing. Another challenge is to study or combine retinal prostheses with neuromodulating drugs, genetic modifications, gene-based cell therapy or optogenetics to optimize the effect of retinal stimulation [10].

## Conclusions

Overall, we aim at establishing the in vitro and in vivo biocompatibility profile of the newly developed OPTO-EPIRET stimulator and showing the surgical feasibility of the implantation in an in vivo setting. At first, a good biocompatibility profile in vitro without any signs of cytotoxicity was demonstrated together with no relevant changes in the expression profile of different essential genes. Secondly, the surgical procedure was shown to be feasible. Nevertheless, the big and stiff array was difficult to implant and to fixate and, hence, vitreal hemorrhage or retinal tearing occurred as main adverse effects. It is also necessary to mention, that transient corneal edema caused difficulties in postoperative imaging. Summarizing the in vivo experiments, no signs of cytotoxicity an no increase of inflammatory CD45-positive cells were detected. However, immunohistological investigations showed an increased glial reaction, increased eosinophilia, and GFAP activity in the retinal tissue beneath the implanted device.

To conclude, the biocompatibility profile and surgical feasibility were characterized in detail and the detected adverse events can be used as guidance for further surgical procedures. It was shown that implanting a huge and complex array epiretinally features risks and is still a challenge that has to be accomplished satisfactorily. The safety profile in terms of intra- and postoperative complications has to be improved by further enhancing design details of the implant such as more flexibility, less stiffness, optimized fixation. Future experiments will comprise the functional analysis of the OPTO-EPIRET stimulator by implanting active devices in an acute in vivo setting. The implantation of a functional, fully intraocular OPTO-EPIRET device would reduce the risk of severe infections as no extraocular components or connections are no longer needed and the OPTO-EPIRET could integrate targeted eye movements to optimize the visual information.

## Materials and methods

### Concept/fabricating of the structures

Description and testing of the electrical properties of the OPTO-EPIRET array were published previously [15].

**Fig. 8:**
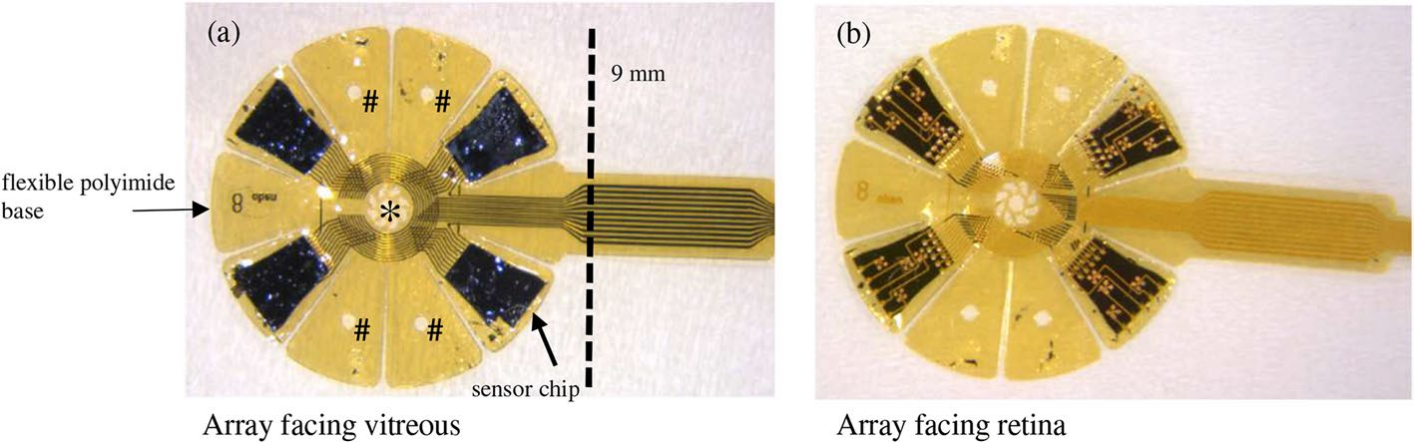
The OPTO-EPIRET array. **a** Photograph of the OPTO-EPIRET array, view on the vitreous facing side. The dashed line illustrates the diameter of the array. Also, the implanted dummy array was cut at this position. **b** Photograph of the OPTO-EPIRET array, view on the retinal facing side. Asterisk: central aperture for the retinal tack; number sign: peripheral aperture for the retinal tack

In brief, the new implant consists of a flexible polyimide carrier foil coated with the hydrophobic and biocompatible parylene C for insulation. The device was slightly pre-curved to better fit the bulb and has a diameter of 9 mm, consisting of nine identical wings and a tenth wing, which serves as the connecting cable for the conductor tracks (see Fig. 8). Thus, the OPTO-EPIRET covers approximately 64 mm^2^ of the retinal surface, which represents a visual angle of approximately 30° [14, 35]. In the middle and on each wing of the array, there are apertures to fixate the implant on the retinal surface with a retinal tack (Geuder AG, Heidelberg, Germany). Four independent ASICs were mounted on four separated wings. Each IC includes a set of integrated image sensor with photodiodes, a signal processing unit, and a constant current stimulator device, which can electrically evoke neuronal action potentials. The chips were thinned. By this, the silicon-based devices reached a certain flexibility and, additionally, it was possible to illuminate the photodiodes from the backside of the chips. The scaffolds were manufactured by ourselves (Institute of Materials in Electrical Engineering (IWE1), RWTH Aachen University; Department of Electronic Components and Circuits, University Duisburg-Essen; Fraunhofer Institute IMS in Duisburg). The ASICs were flip-chip bonded on the polyimide carrier foil. Four gold electrodes were linked with one ASIC, in total 16 electrodes on one array. The electrodes were coated with platinum and sputtered with iridium oxide to reduce the electrode impedance, as it was already done for the VLARS array [12].

### In vitro biocompatibility

Cell culture, in vitro biocompatibility testings and quantitative real-time polymerase chain reaction (qRT-PCR) were performed as previously published [16] and described in standard DIN ISO 10993 “biological evaluation of medical devices”, parts 5 and 12. The analyses were separately performed with the flexible polyimid carrier foil (called flexible polyimide base) and the ASICs (called sensor chips).

#### Cell culture

Cell culture was performed as previously reported [16]. In brief, L-929 cells (ATCC No. CCL-1) were cultivated in minimum essential medium (MEM) with Earle’s salts (Biochrom, Berlin, Germany) supplemented with 200 mM l-glutamine (Sigma Aldrich Chemie, Steinheim, Germany), 10% fetal bovine serum (FBS; PAN-Biotech, Aidenbach, Germany), 80 U/mL penicillin (Sigma Aldrich Chemie), and 80 µg/mL streptomycin (Sigma Aldrich Chemie), and maintained at 37 °C in a humidified atmosphere of 95% air and 5% CO_2_. R28 cells were cultivated in Dulbecco’s modified Eagle’s medium (DMEM; Biochrom) supplemented with 10% FBS, 5.5 mL of 100 × MEM vitamins (Gibco, Paisley, UK) and nonessential amino acids (Biochrom), 80 U/mL penicillin, and 80 µg/mL streptomycin and maintained at 37 °C in humidified atmosphere of 95% air and 5% CO_2_. Medium was changed three times a week. Cells were subdivided once a week at a ratio of 1:20 [16].

#### Indirect contact with flexible polyimide bases and sensor chips

Cytotoxicity analysis of nondirect contact was performed as previously described [16]. In brief, L-929 and R28 cells were incubated with extractive media of the tested materials in different dilutions (1:1, 1:2, 1:4). The test materials were analyzed and compared to different reference materials (RM, Hatano Research Institute, Hadano, Japan) with defined levels of cytotoxicity (RM A: moderate cytotoxity, polyurethane film containing 0.1% zinc diethyldithiocarbamate; RM B: weak cytotoxicity, 0.25% zinc dibutyldithiocarbamate; RM C: no cytotoxicity, high-density polyethylene film). Glass was used as internal negative control. After 24 h, a luminescent cell viability assay was performed (CellTiter-Glo^R^; Promega, Madison, WI) according to the manufacturer’s protocol. Each sample was measured in triplicate and the mean value was used for further analysis.

#### Direct contact with flexible polyimide bases and sensor chips

Cytotoxicity analysis of direct contact was performed as previously described [16]. In brief, L-929 and R28 cells were cultivated on the test materials (flexible polyimide base; silicon and silicon nitride-based CMOS sensor chips) and on glass (no-substrate control) at a density of 31,250 cells/cm^2^ for L929 cells and 10,000 cells/cm^2^ for R28 cells. The testings were performed without encapsulation of the test materials to evaluate a possible cytotoxicity of the basic materials themselves. After 72 h, a live/dead cell staining assay with fluorescein diacetate (FDA, Sigma-Aldrich Chemie), and propidium iodide (PI, Sigma-Aldrich Chemie) was performed. Fluorescence microscopy allowed for differentiation between vital (green FDA staining) and dead (red PI staining) L-929 cells.

#### Quantitative real-time polymerase chain reaction (qRT-PCR)

Analysis of the gene expression profile of cultivated R28 cells was performed as previously described [16]. In brief, qRT-PCR was conducted to analyze the expression profile of different specific genes involved in cell cycle and representing retinal or neuronal/glial markers: MYC, CCNC, TP53, NRP1, S100B, VIM, CDH2 together with the house-keeping genes GAPDH (glyceraldehyde-3-phosphate dehydrogenase) and HPRT1 (hypoxanthine–guanine phosphoribosyltransferase).

R28 cells were plated on glass and on the different test structures. After 72 h at 37 °C, cultivation was terminated. Total ribonucleic acid (RNA) was isolated using the RNeasy Mini Kit together with the RNase-free DNase Set (Qiagen, Hilden, Germany) according to the manufacturer’s protocol, and reverse transcription was carried out on 20 ng total RNA using the Reverse Transcription System (Promega, Madison, WI). Real-time qPCR was performed on a LightCycler 1.2 Instrument using the LightCycler FastStart DNA Master SYBR Green I kit (Roche Diagnostics, Mannheim, Germany) according to the manufacturer’s recommendations. The cNDA samples were run in duplicate using the following primers: GAPDH (GenBank Accession: X02231, F: 5′-TGG GAA GCT GGT CAT CAA C-3′ and R: 5′-GCA TCA CCC CAT TTG ATG TT-3′), HPRT1 (GenBank Accession: M63983, F: 5′-CTC CTC AGA CCG CTT TTC C-3′ and R: 5′-TCA TAA CCT GGT TCA TCA TCA CTA A-3’), VIM (GenBank Accession: X62952, F: 5′-AAC ACT CCT GAT TAA GAC GGT TG-3′ and R: 5′-TCA TCG TGG TGC TGA GAA GT-3′), NRP1 (GenBank Accession: AF016296, F: 5′-CAT AGT GGG CTC GGA CTG A-3′ and R: 5′-GGT CCA GCT GTA GGC ACT TC-3′), CDH2 (GenBank Accession: AF097593, F: 5′-CCA TCA TCG CGA TAC TTC TG-3′ and R: 5′-CCA TAC CAC GAA CAT GAG GA-3′), S100B (GenBank Accession: J03627, F: 5′-AAG GGA GTT CCC TGG GTT T-3′ and R: 5′-CAC TGG TCC AGG TCT TTC ATT-3′), CCNC (GenBank Accession: NM_001100472, F: 5′-AAA ACC ACC TCC GAA CAG TG-3′ and R: 5′-GAT TGG CTG TAG CTA GAG TTC TGA C-3′), MYC (GenBank Accession: NM_012603, F: 5′-GCT CCT CGC GTT ATT TGA AG-3′ and R: 5′-GCA TCG TCG TGA CTG TCG-3′), and TP53 (GenBank Accession: X13058, F: 5′-AGA GAG CAC TGC CCA CCA-3′ and R: 5′-AAC ATC TCG AAG CGC TCA C-3′). Reactions were performed with diluted complementary deoxyribonucleic acid (cDNA) corresponding to 0.4 ng of initially used total RNA and a primer concentration of 0.10 µM and 0.25 µM, respectively. Thermal cycler conditions were as follows: initial denaturation at 95 °C for 10 min, followed by 50 cycles with denaturation at 95 °C for 10 s, annealing at 60 °C for 8 s, and elongation at 72 °C for 15 s. Melting curve analysis confirmed the amplification specificity of each primer pair. Data were processed with LightCycler software 3.5.3 and evaluated using the comparative CT (2^−ΔΔCT^) method, which describes relative gene expression [44]. Even though analysis of the data revealed a constant expression for both internal control genes, the lowest standard deviation was achieved with HPRT1. Thus, gene expression levels of all target genes were normalized to the HPRT1 expression level.

### In vivo biocompatibility

Implantation surgery and follow-up examinations were performed as previously published [12].

#### Device handling and implantation in cadaveric rabbit eyes

Before performing the implantation surgery with inactive arrays in rabbits, the feasibility was tested in cadaveric rabbit eyes (obtained from the Institute of Laboratory Animal Science, RWTH Aachen University, Germany and from a local breader). A surgical microscope was used for surgery (Zeiss Model OPMI 6-CFR VX, S5 Tripod, Carl Zeiss AG, Jena, Germany). For the feasibility testing, the inactive arrays were not fixated at the posterior pole.

#### Performing surgery in rabbit eyes

All animal experiments were performed according to the declaration of the association for research in vision and ophthalmology (ARVO) for the use of animals in research, the German Law for the Protection of Animals as well as the guidelines of the federation of European laboratory animal science association (FELASA) after approval was obtained by the regulatory authorities (84-02.04.2016.A412). Efforts were made to reduce the number of experimental animals and their suffering. Six female chinchilla bastard rabbits (2294 ± 269 g) were housed under standard conditions with 12 h light/dark cycle and access to water and food ad libidum. The implantation was performed on the right eye following the protocol established with cadaveric rabbit eyes. The left eye served as a control.

Before performing surgery, proxymetacaine hydrochloride 0.5% eye drops (Proparakain-POS, Ursapharm, Saarbrücken, Germany) for local anesthesia and hydrochloride 2.5% and tropicamide 0.5% eye drops to dilate the pupil (MS-mydriatic eye drops, Pharmacy of the University Hospital RWTH Aachen, Germany) were applied to the right eye. Afterwards, the animals were anesthetized with medetomidine (0.3 mg/kg bodyweight, Domitor®, Orion Corporation, Espoo, Finland) and ketamine (0.02 g/kg bodyweight, MEDISTAR Arzneimittelvertrieb GmbH, Ascheberg, Germany) subcutaneously. The anesthesia was maintained with isoflurane gas (Forene®, AbbVie, Ludwigshafen, Germany) after endotracheal intubation and the rabbits received fentanyl (0.1–1 mL/h, ROTEXMEDICA, Trittau, Germany) intravenously to prevent pain.

The surgical field was disinfected with 10% povidone–iodine solution (Betaisodonna, Mundipharma GmbH, Limburg, Germany). After sterilizing the implant by placing it for 10 s into 70% ethanol (Pharmacy of the University Hospital RWTH Aachen, Germany) and preparing sterile conditions, a canthotomy was performed. Afterwards, the nictitating membrane was removed and the conjunctiva was opened. The rectus muscles were hooked and looped with polyester threads (Mersilene 4-0, Ethicon LLC, San Lorenzo, USA). Three 23 gauge ports were placed with 1.5 mm distance to the limbus into the pars plana for vitrectomy for infusion, light source and surgical instruments (Fritz Ruck Ophthalmologische Systeme, Eschweiler, Germany). The next steps were an anterior capsulorhexis (see Fig. 4a) and the lensectomy (see Fig. 4b), which was performed with a standard phacoemulsification technique (OMNI, Fritz Ruck Ophthalmologische Systeme, Eschweiler, Germany). The posterior capsule was removed (see Fig. 4c) and a complete vitrectomy was performed (see Fig. 4d). The eye was filled with PFCL (F-Decalin 1.93 g/cm Fluoron GmbH, Ulm, Germany). Through a corneal access, the array was placed in the anterior chamber. By using a push–pull instrument (Geuder GmbH, Heidelberg, Germany) it was forwarded to the vitreous cavity. While implanting the array, the PFCL was removed slowly. After tackling the array, the bulb was filled with air at the pressure of 25 mmHg and all cuts (the ports, the corneal incision and the conjunctiva) were sutured consecutively.

At the end of the surgery, 750 mg cefuroxime (Cefuroxim Fresenius 750 mg, Fresenius Kabi DE, Bad Homburg, Germany) and 4 mg dexamethasone-dihydrogenphosphate-dinatrium (Fortecortin Inject 4 mg, Merck KGaA, Darmstadt, Germany) were injected into the anterior chamber and a subconjunctival injection of 8 mg gentamicin (Gentamicin 8 mg Rotexmedica, ROTEXMEDICA GmbH Arzneimittelwerk) and 50 mg prednisolon-21-succinat (Prednisolon H 50 mg, Merck) was applied to reduce inflammation and to prevent infection.

After the surgery, the animals received subcutaneously 5 mg/kg bodyweight carprofen (non-steroidal anti-inflammatory drug, Rimadyl 20 mg, Zoetis GmbH, Berlin, Germany) once a day for 3 days. Anti-inflammatory and antibiotic eye drops and ointment (Isopto-Max, Dexamethason 1 mg/g, Neomycin 3500 IE/g, Polymyxin-B-sulfat 6000 IE/g, Novartis Pharma, Basel, Switzerland) were applied topically directly after surgery and for at least 1 week. In some cases, the topical treatment containing corticosteroids was extended for some weeks, depending on the presence of inflammation or in case of corneal edema.

#### Follow-up examinations

The rabbits were daily examined by checking behavior. Eye drops were reduced over the time according to the level of irritation. The follow-up examinations were performed over a time period of 12 weeks at defined time points (week 1, 2, 4, 8, 12), similar to evaluation of the VLARS array [12]. Due to time constraints the follow-up examination at week 8 was shifted to the following week in 3 cases. For follow-up slit-lamp examination, fundoscopy, OCT imaging, ultrasound imaging and fundus photography were performed under general anesthesia with ketamine and medetomidine.

Slit-lamp examination and fundoscopy: For clinical evaluation a portable ophthalmoscope (Keeler Spectra IRIS, Keeler ltd., Berkshire, United Kingdom) was used in combination with a 20D (diopter) lens (Volk Optical Inc., Mentor, USA). To evaluate signs of infection or inflammation corneal clarity, presence of fibrin or hyphema in the anterior chamber and vitreous clarity were assessed. Furthermore, the position and fixation of the array were checked.

OCT imaging: Spectral-domain (SD) OCT images and infrared images were taken in the periphery and in the center of the array with a Spectralis OCT system (Heidelberg Engineering, Heidelberg, Germany).

Fundus photography: A Zeiss FF450Plus camera system (Carl Zeiss AG, Jena, Germany) with a Canon EOS 5D digital camera capturing system (Canon Inc., Tokyo, Japan) was used. In cases of good visibility, the array’s position was captured by photography.

Ultrasound imaging: In case of severe corneal edema or vitreous hemorrhage ultrasound imaging was performed to evaluate the position of the array, its fixation, and the presence or absence of retinal tearing or detachment. Images were taken with a 10 MHz B-scanning probe (Aviso S, Quantel Medical, Cournon d’Auvergne Cedex, France).

#### Histology

At the end of the last follow-up examination, which was performed under general anesthesia with ketamine and medetomidine, the animals were killed with an overdose of 2 mL/kg bodyweight pentobarbital-sodium (narcoren, 160 mg/mL, Boehringer Ingelheim, Ingelheim am Rhein, Germany). The rabbit eyes were dissected and the bulbs were fixated in Methacarn fixative [consisting of methanol (Roth, Karlsruhe, Germany), chloroform (VWR, Darmstadt, Germany) and glacial acetic acid (Roth) at a ratio of 6:3:1] at room temperature for 24 h. The bulbs were dehydrated in ethanol and encapsulated in paraffin (Sakura, Staufen, Germany). Sections of 5 µm were cut and stained with hamatoxylin (Mayers Hämalaun, AppliChem, Darmstadt, Germany) and eosin (Roth) (H&E) according to standard procedures. For immunohistochemistry, sections were treated with xylol (Fischar, Saarbrücken, Germany) and rehydrated. After blocking the endogenous peroxidase, the sections were washed with phosphate-buffered saline (PBS, Biochrom) and incubated with normal horse serum (Vector Kit MP-7402, Vector Laboratories, Burlingame, USA) for 20 min. The sections were incubated for one hour with the primary antibodies against GFAP (MAB360, Merck) or CD45 (MCA808GA, Bio-Rad Laboratories, Feldkirchen, Germany), washed with PBS, and incubated with the secondary antibody (Vector Kit MP-7402, Vector Laboratories) for 30 min. After washing with PBS, the sections were incubated with ImmPACT DAB (Vector SK 4105, Vector Laboratories) for 8 min. Finally, the sections were dehydrated and encapsulated in xylol. Every step of the staining procedure occurred at room temperature. For negative controls, sections were treated as described, but without the use of primary antibodies.

### Statistical analysis

All statistical analyses were performed with GraphPad Prism (version 7, San Diego, CA, USA). All data are calculated as mean ± standard deviation (SD). One sample two-tailed t-test, unpaired two-tailed *t*-test, and one-way ANOVA with post hoc Dunnett test were performed as detailed within the figure legends.

## Data Availability

The datasets used and/or analyzed during the current study are available from the corresponding author on reasonable request.

## Abbreviations

ARVO: Association for research in vision and ophthalmology
ASIC: Application Specific Integrated Circuit
CCNC: Cyclin-C
CDH2: Cadherin-2
cDNA: Complementary deoxyribonucleic acid
CMOS: Complementary metal–oxide–semiconductor
CO_2_: Carbon dioxide
d: Day
D: Diopter
DMEM: Dulbecco’s modified Eagle’s medium
e.g.: For example
FBS: Fetal bovine serum
FDA: Fluorescein diacetate
FELASA: Federation of European laboratory animal science association
GAPDH: Glyceraldehyde-3-phosphate dehydrogenase
GCL: Ganglion cell layer
GFAP: Glial fibrillary acidic protein
HPRT1: Hypoxanthine–guanine phosphoribosyltransferase
H&E: Hematoxylin and eosin
IC: Integrated Circuit
IOP: Intraocular pressure
INL: Inner nuclear layer
IPL: Inner plexiform layer
MEM: Minimum essential medium
MYC: Myc proto-oncogene protein
*n*, no.: Number
NRP-1: Neuropilin-1
OCT: Optical coherence tomography
ONL: Outer nuclear layer
OPL: Outer plexiform layer
OPTO-EPIRET: Epiretinal implant with integrated sensor chips for optical capturing
PBS: Phosphate-buffered saline
PCR: Polymerase chain reaction
PFCL: Perfluorocarbon liquid
PW: Propidium iodide
PR: Photoreceptors
qRT-PCR: Quantitative real-time polymerase chain reaction
RGC: Retinal ganglion cells
RM: Reference material
RNA: Ribonucleic acid
RNFL: Retinal nerve fiber layer
RP: Retinitis pigmentosa
RPE: Retinal pigment epithelium
SD: Standard deviation
SD-OCT: Spectral-domain optical coherence tomography
S100B: Protein S100-A10
TP53: Cellular tumor antigen p53
US: Ultrasound
VIM: Vimentin
VLARS: Very large electrode array for retinal stimulation
vs.: Versus
